# Delusional themes and forensic status in schizophrenia spectrum disorders: a machine learning based discriminative study

**DOI:** 10.3389/fpsyg.2026.1840906

**Published:** 2026-07-22

**Authors:** Amr A. N. Eleryan, Friederike Boudriot, Marc Dörner, Andreas B. Hofmann, Lena Machetanz, Johannes Kirchebner

**Affiliations:** 1 University of Zurich, Zürich, Switzerland; 2 Department of Forensic Psychiatry and Psychotherapy, Psychiatrische Dienste Aargau, Windisch, Switzerland; 3 Department of Consultation-Liaison-Psychiatry and Psychosomatic Medicine, University Hospital Zurich, University of Zurich, Zurich, Switzerland; 4 Department of Neurology, University Hospital Zurich, University of Zurich, Zurich, Switzerland; 5 German Center for Neurodegenerative Diseases (DZNE) within the Helmholtz Association, Magdeburg, Germany; 6 Adult Psychiatry and Psychotherapy, University Hospital of Psychiatry Zurich, University of Zurich, Zurich, Switzerland; 7 Forensic Psychiatry and Psychotherapy, University Hospital of Psychiatry Zurich, University of Zurich, Zurich, Switzerland; 8 University Hospital of Forensic Psychiatry and Psychology, University of Bern, Bern, Switzerland

**Keywords:** criminal behavior, delusions, machine learning, psychopathology, schizophrenia spectrum disorders

## Abstract

**Background:**

Schizophrenia spectrum disorders (SSD) are associated with an elevated risk of violent and offending behavior; however, the specific contribution of delusional content remains insufficiently understood. Prior research has yielded inconsistent findings regarding the relationship between particular psychopathological symptoms and delusional themes and criminal behavior. A better understanding of symptom-specific phenomenological differences between forensic psychiatric (FPP) and general psychiatric patients (GPP) may inform clinical assessment in forensic settings. This study therefore aimed to examine whether specific psychopathological symptoms discriminate between FPP and GPP with SSD.

**Methods:**

In this retrospective study, data from 740 patients with SSD (370 FPP, 370 GPP) treated at a Swiss university hospital were analyzed. From an initial dataset comprising over 500 variables, 103 symptom-focused variables were selected to isolate psychopathological characteristics. Following preprocessing, the dataset was split into training and validation subsets. After the application of dimension reduction techniques, multiple supervised machine learning algorithms were applied and compared using nested cross-validation. Model performance was assessed using area under the curve (AUC), balanced accuracy, sensitivity, and specificity. A *post hoc* analysis further examined the impact of comorbid substance use and personality disorders.

**Results:**

Naïve Bayes achieved the best performance (AUC = 0.81, balanced accuracy = 78.2%), with stable results in the validation sample. Two variables emerged as key discriminators: bizarre delusions (46% in FPP vs. 2% in GPP) and aggressive delusional content (69% vs. 19%). Global symptom severity measures, including PANSS scores, did not contribute to the model. The *post hoc* analysis resulted in only minimal changes, with estimates remaining within the original confidence intervals, indicating robust model stability.

**Conclusion:**

The qualitative content of delusions – particularly bizarre and aggressive themes – shows substantial discriminative value for forensic status in SSD, beyond overall symptom severity and established comorbid risk factors. These findings highlight the relevance of symptom-specific, cognitive-affective mechanisms in forensic evaluations. Incorporating structured assessment of delusional content may complement existing psychometric approaches in forensic psychiatric evaluations.

## 1 Introduction

Schizophrenia spectrum disorders (SSD) comprise a group of chronic and severe mental illnesses characterized by disruptions in cognition, emotion, and perception. While inherent symptoms – especially delusions and hallucinations – are linked to violent behavior, not all individuals with SSD engage in violent acts. The risk factors for violence remain complex and multifactorial. Among delusions, persecutory, control, and grandiose contents are significant contributors to violent behavior (Swanson et al., 1990; Fazel et al., 2009a). Aggression in individuals with SSD can be attributed to a complex interplay of factors. Substance use disorders are a significant contributor, as they can exacerbate psychiatric symptoms and lower inhibitions, leading to impulsive and aggressive behavior (Fazel et al., 2009b; Witt et al., 2013). In general, substance abuse can intensify delusional thinking and hallucinations. Additionally, drug abuse can impair judgment and reduce the effectiveness of antipsychotic treatments, further contributing to the risk of violence and self-harm (Swartz et al., 1998).

Delusions, defined as fixed false beliefs not grounded in reality, are a core symptom of schizophrenia. They significantly impair an individual’s level of functionality. Persecutory delusions are associated with defensive aggression (Cheung et al., 1997; Coid et al., 2013; Witt et al., 2013; Grohmann et al., 2024). Likewise, delusions of control or command hallucinations, where the individual feels coerced into violent actions, are strongly linked to violent behavior (Cheung et al., 1997). Grandiose delusions, involving an inflated sense of one’s abilities or importance, potentially lead to either defensive or offensive violence (Swanson et al., 1990) (Wallace et al., 2004; Witt et al., 2013). Conversely, some research found no significant connection between delusional themes and aggression (Douglas et al., 2009). These mixed results underscore the importance of examining the specific content of delusions when assessing the risk of violence in patients with SSD and highlight the need for more research to clarify these relationships.

A systematic review conducted in 2016 specifically examined the relationship between paranoia and aggression in psychotic patients. The analysis found a significant correlation between paranoid delusions and an increased risk of aggression. According to the findings, the severity of paranoid symptoms was directly related to the likelihood of aggressive behavior, particularly when patients perceived external threats or attacks (Darrell-Berry et al., 2016).

Apart from positive symptoms and substance use disorders, individual differences, additional psychiatric symptoms, and social isolation play a critical role in the occurrence of violent behavior and must be considered during clinical assessments and treatment planning (Bo et al., 2011; Darrell-Berry et al., 2016). Medication non-adherence, a common challenge in treating schizophrenia, can further exacerbate delusions and increase the risk of violent behavior (Swartz et al., 1998).

Addressing factors such as medication adherence, social support, and substance use can further mitigate the risk of violence and improve overall outcomes for those with schizophrenia (Kirchebner et al., 2023; Bender et al., 2024).

Recent advances in machine learning (ML) provide promising tools with discriminative value between forensic and non-forensic individuals with SSD based on clinical and socio-environmental factors. When properly validated, ML based risk assessments may improve the management of adverse events in general and violence risk in particular (Hofmann et al., 2025).

While differences between violent and non-violent offenders diagnosed with SSD have been studied (Grohmann et al., 2024), research covering the occurrence of distinct delusional themes in general psychiatric (GPP) and forensic psychiatric patients (FPP) remains sparse. The aim of this study is therefore to examine psychopathological differences between FPP and GPP, including delusional content, hallucinations, and symptom severity measures. Specifically, the study seeks to identify which psychopathological variables differentiate forensic from general psychiatric patients, utilizing supervised machine learning algorithms to analyze complex interplays between a myriad of variables. Thereby, this research seeks to characterize phenomenological differences between these clinical populations and to identify which psychopathology is associated with forensic psychiatric status.

## 2 Materials and methods

### 2.1 Design and study population

This study is a retrospective analysis involving a total of 740 patients, all diagnosed with any SSD as specified in ICD-9 (for earlier cases) and ICD-10 (for more recent cases) (Slee, 1978; WHO, 1992). The study cohort consists of two groups: 370 patients who received forensic treatment and 370 patients who received general psychiatric treatment. All participants were treated at the University Hospital of Psychiatry Zurich, Switzerland. The forensic patients (referred to as Forensic Psychiatric Patients or FPP) were treated at the Center for Inpatient Forensic Therapy, which is part of the Department of Forensic Psychiatry. The general psychiatric patients (referred to as General Psychiatric Patients or GPP) were treated at the Center for Integrative Psychiatry, which falls under the Department of Adult Psychiatry. The GPP group was chosen as a suitable comparison group for this study since these typically comprise chronic cases with significant impairments in psychosocial functioning and psychopathology, as well as chronic courses of disorders. FPP were admitted to treatment based on court decisions due to a lack of culpability under Article 59 of the Swiss Criminal Code or admitted for crisis intervention during imprisonment. GPP were admitted either voluntarily or compulsorily, and were matched for sex and year of admission.

Importantly, each patient was included only once and evaluated over a single treatment episode, defined as the period from admission to discharge. For patients who had several admissions in their history, the most recent hospitalization was used as referenced case. Accordingly, there were no multiple admissions per patient considered in the dataset. All FPP included in the study were admitted between 1982 and 2016. Given the legally mandated long duration of inpatient forensic measures – typically lasting several years, with a minimum duration of 5 years under Swiss criminal law – and the resulting low turnover, the inclusion period for FPP necessarily spanned multiple decades. This ensured the recruitment of a sufficiently large and representative forensic sample despite the limited annual number of new admissions.

### 2.2 Data source

Data were retrospectively independently two senior psychiatrists extracted from electronic health records by two senior psychiatrists through directed qualitative content analysis (Assarroudi et al., 2018). The extraction protocol was developed based on established forensic psychiatric assessment criteria and refined through iterative consensus discussions involving the chief psychiatrist and chief psychologist (Seifert and Leygraf, 1997). Following protocol development, initial coding was performed on a pilot sample (approximately 5% of cases) to test operationalization. The coding framework was then finalized with specific definitions and examples to ensure consistent application. To assess reliability, 10% of all cases were independently coded by a second trained rater. Inter-rater reliability was substantial, with Cohen’s Kappa = 0.78 (Brennan and Hays, 1992). Disagreements between raters were resolved through structured consensus discussions, with consultation from a third senior clinician when immediate agreement could not be reached. Given the nature of the data sources (FPP records include police and court documents unavailable for GPP), complete blinding to group membership was not feasible. However, raters remained blinded to the specific research hypotheses to minimize bias. Data sources included detailed medical records, clinical assessments, and, for forensic patients, additional legal documentation including police reports, court proceedings, and testimony records. In order to collect a sufficient amount of data for machine learning purposes, treatments between 1982 and 2016 were included. The entire dataset comprises more than 500 variables, having facilitated a large variety of former exploratory analyses. For the present analysis, we deliberately selected 103 variables from several domains focusing exclusively on symptomatology. This approach was chosen to isolate the specific contribution of psychopathological features without the confounding influence of possible risk factors such as substance use or personality disorders. These domains included patient history, diagnoses, type of delusion, Positive and Negative Syndrome Scale (PANSS) sum score and single items upon admission and discharge (modified to a three-tier system, with the sum score ranging from 0 to 60) (Kay et al., 1987). Delusional content categories were not coded as mutually exclusive. Accordingly, the same delusional belief could be assigned to more than one category when appropriate. Aggressive content was coded as an additional content dimension rather than as an alternative to other subtypes. Thus, persecutory or poisoning delusions involving threats of harm, violence, assault, killing, torture, or poisoning were coded both according to their specific delusional subtype and as aggressive delusional content. By contrast, persecutory beliefs without violent or harmful content were not coded as aggressive delusional content.

The full list of variables is provided in the Supplementary material. The number of variables was reduced due to the relative paucity of observations, thereby reducing the risk of overfitting, which is a major pitfall in machine learning (Ying, 2019; Charilaou and Battat, 2022).

### 2.3 Statistical analysis

ML was employed in several steps. First, data preparation involved the omission of variables with more than 33% of missing values and the definition of predictor and outcome variables, with the latter dichotomized as “Is the patient not forensic? – Yes/No”. The 33% missing data exclusion threshold was chosen as a conservative approach to maintain data quality and model stability. While there are no universally established cut-offs for acceptable missing data rates (Schafer and Graham, 2002), we selected this threshold based on our dataset characteristics to balance variable retention with statistical reliability. A variable was considered missing if no information regarding the respective feature could be identified in the available clinical documentation. This includes cases where a specific symptom (e.g., a particular delusional theme) was not mentioned in the records and could not be inferred with sufficient certainty. Importantly, the absence of a symptom was not assumed if it was not explicitly documented. Rather, such cases were coded as missing to avoid introducing bias through implicit negative assumptions.

The dataset was then split into two subsets, with 70% allocated for training and 30% for validation. This split follows established machine learning practices, providing sufficient training data for model development while maintaining adequate validation samples for unbiased performance assessment (Xu and Goodacre, 2018; Joseph, 2022). Missing values in the training subset were imputed using the mean for continuous variables and the mode for categorical variables. No upsampling was performed since both groups are equally large. Dimension reduction was carried out by using a random forest algorithm based on mean decrease in accuracy as the importance measure.

A threshold-based strategy was applied here to retain only variables that contributed meaningfully to model performance. Consistent with established principles of feature selection, variables were removed iteratively in a stepwise fashion, retaining only those whose inclusion improved the AUC by more than 5%. Once the increase in AUC dropped below this predefined threshold, additional variables were no longer considered beneficial, as they were unlikely to improve model performance and could instead introduce unnecessary complexity and increase the risk of overfitting.

**Figure 1:**
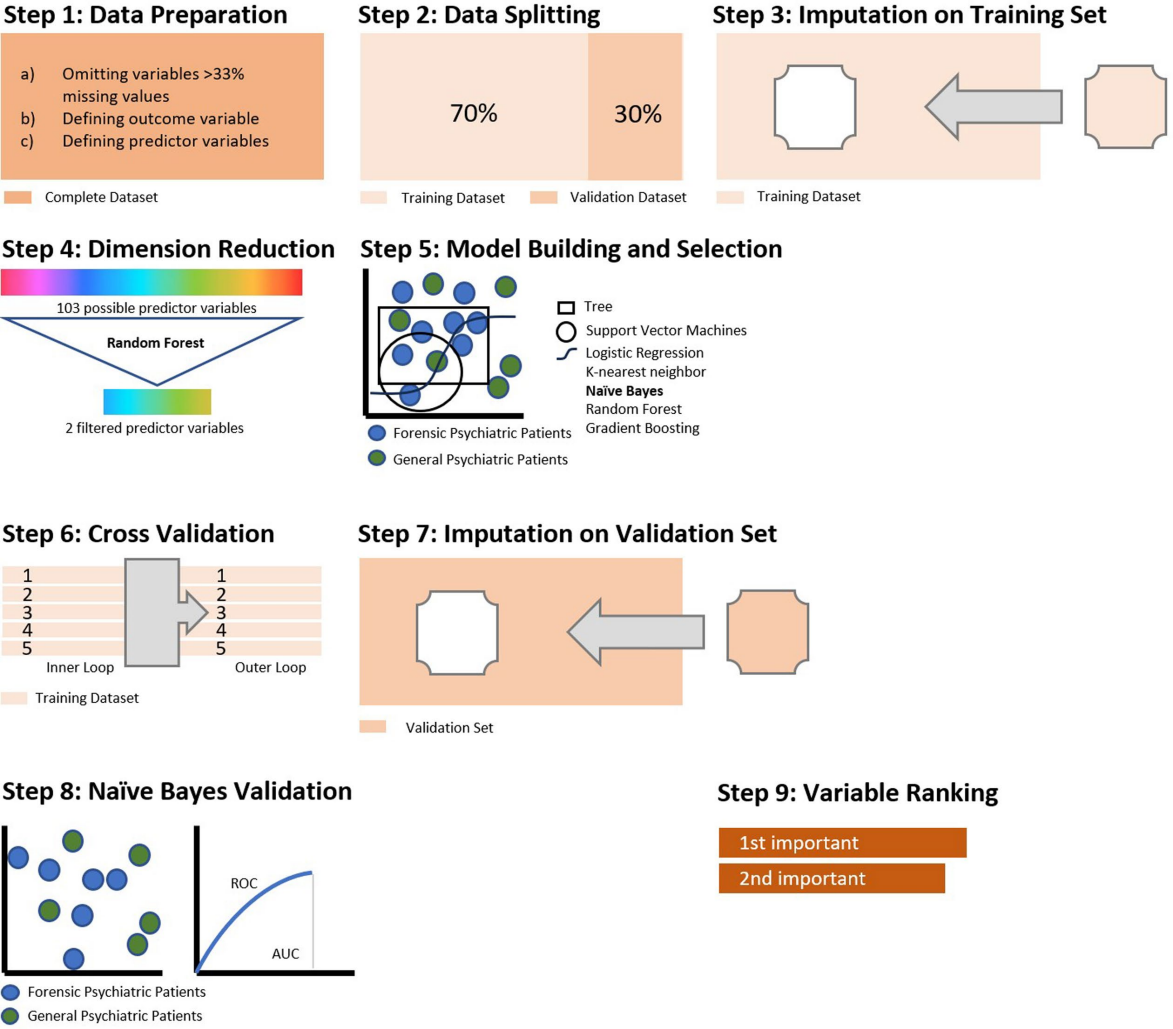
Data preparation, machine learning model testing and validation.

This led to a reduction of variables *r* from 103 to 2. Several machine learning algorithms were applied to the training subset, including Naïve Bayes, Logistic Regression, Decision Tree, Random Forest, Gradient Boosting, K-Nearest Neighbor, and Support Vector Machines. Nested cross-validation was conducted to ensure the reliability and generalizability of the models. The algorithm with the best performance parameters, as indicated by the AUC as a parameter of goodness of fit and level of discrimination, sensitivity, specificity, balanced accuracy (incorporating sensitivity and specificity) as well as positive and negative predictive value, was chosen. Accordingly, Naïve Bayes was selected for the further steps. The Naïve Bayes algorithm was then validated using the still unused validation dataset, with missing values imputed similarly to the training subset. The final model’s statistical performance was calculated using the previously stated measures. In a final step, the variables were ranked based on their relevance for the model. Descriptive statistics were used for basic group comparisons, including means with standard deviations and proportions. To assess temporal consistency, we conducted a sensitivity analysis restricting the dataset to cases from 2000 to 2016, applying the identical final ML algorithm to evaluate model stability across the study period. Moreover, as basic descriptive data (see result section Table 1) revealed different prevalence of substance use and personality disorder, a *post hoc* sensitivity analysis to address potential confounding was conducted. Thus, we repeated the final model validation including substance use disorders and personality disorders as additional predictors (4 variables total), using identical validation procedures. More specifically, the items that were found to be influential to the final model were combined with the following two: presence of any substance consumption pattern corresponding to the ICD-10 diagnosis F1x.1 or F1x.2 (excluding F10) and any personality disorder listed in the ICD-10 (F60.X, F61) and/or the DSM-V. These four variables were then run on the final model. The software used for the analysis included R version 3.6.3 (R Project), the MLR package v2.171 (Bischl, Munich, Germany), and MATLAB R2019a (MATLAB and Statistics Toolbox Release 2012). Figure 1 displays all machine learning steps.

**Table 1:** Basic sample characteristics.

Variable description	Forensic psychiatric patients		General psychiatric patients	
	*n*/*N* (%)	Mean (SD)	*n*/*N*(%)	Mean (SD)
Age upon admission	34.2 (10.2)		36.2 (12.2)	
Sex*: male	339/370 (91.6)		339/370 (91.6)	
Country of birth: Switzerland	167/370 (45.1)		245/367 (66.8)	
Civil status: single	297/364 (81.6)		282/364 (77.5)	
Diagnosis: paranoid schizophrenia	294/370 (79.5)		287/370 (77.6)	
Comorbid substance use disorder	269/370 (72.7)		183/327 (56.0)	
Comorbid personality disorder	47/370 (12.7)		26/370 (7.0)	

SD, standard deviation. *According to patients’ files.

ML was chosen over traditional statistical approaches due to our high-dimensional dataset with 103 variables and the exploratory nature of our research question (James et al., 2013). Traditional statistical analysis typically requires *a priori* variable selection and hypothesis formulation based on researcher assumptions, risking that important predictors are overlooked while irrelevant variables are included. In contrast, ML shifts the focus from testing a-priori hypotheses to discovering patterns and optimizing predictive accuracy through algorithmic learning from data (Shmueli, 2010), allowing for complex, non-linear relationships that may not be anticipated a priori. After dimension reduction to two key variables, we then compared multiple algorithmic approaches - including logistic regression alongside more complex methods like Random Forest and Support Vector Machines - to empirically determine optimal performance. Notably, logistic regression performed well in this reduced variable space, but Naïve Bayes achieved slightly superior discrimination Nested cross-validation provided robust protection against overfitting, crucial for high-dimensional clinical data. While the final two-variable model could theoretically be analyzed with any single method, the ML approach was essential for objectively identifying these key predictors from the initial 103 variable space.

## 3 Results

Basic sample characteristics revealed that FPP and GPP showed similar ages upon admission, and patients of both groups were mostly single. As they had been matched for sex, populations showed a high percentage of male individuals, the majority of patients in both groups were men. Nearly 80% of both groups were diagnosed with paranoid schizophrenia. Differences occurred regarding country of birth as FPP were more less often natives of Switzerland. Moreover, they more frequently presented a comorbid substance use and personality disorder. For detailed percentages, please refer to Table 1.

Due to the different nature of their treatment, length of stay varied greatly between the two groups, with a mean length of stay of 220 weeks for FPP in court-mandated therapy under Art. 59 of the Swiss Criminal code for 143 cases and hospitalizations of up to 3 months for crisis intervention during imprisonment. In comparison, length of stay was naturally much shorter for GPP (mean: 8.8 weeks). 73.8% of the FPP had been in general psychiatric inpatient treatment at some time in their history before entering the forensic psychiatric system. GPP had a lower ratio of previous inpatient treatments (57.1%).

As described in the method section, we applied several machine learning algorithms to the training subset (i.e., 70% of total dataset). A comprehensive overview of statistical performances is provided in Table 2. With an AUC of 0.82 and a balanced accuracy of 78,8%, Naïve Bayes outperformed all the remaining algorithms in nearly every aspect, only K-nearest neighbors proved to exhibit a better negative predictive value. Regarding these results, we considered Naïve Bayes as most suitable and therefore chose it for further model validation. When applied to the validation subset, Naïve Bayes exhibited stable performance parameters (see Table 3): sensitivity, i.e., the model’s ability to correctly identify GPP, and specificity, i.e., to correctly identify FPP, were approximately 81% (95% confidence interval (CI) ranging from 72 to 87%) and 76% (95% CI 66–83%), respectively. The AUC (0.81, 95% CI 0.75–0.87) is considered to be good (Nahm, 2022). A sensitivity analysis restricting data to 2000–2016 yielded consistent performance (AUC 0.84, balanced accuracy 81.0%) compared to the full dataset. The *post hoc* analysis adding substance use and personality disorders as predictors showed minimal model changes (AUC 0.82, balanced accuracy 78.2%). Both additional analyses’ results fall within the 95%-CI of the original analysis (see Table 3).

**Table 2:** Machine learning models and performance in nested cross-validation.

Statistical procedure	Balanced accuracy (%)	AUC	Sensitivity (%)	Specificity (%)	PPV (%)	NPV (%)
Logistic regression	77.5	0.81	80.8	74.3	74.6	80.4
Tree	77.4	0.81	80.5	74.4	74.9	80.4
Random forest	77.2	0.80	80.3	74.2	74.5	80.4
Gradient boosting	77.4	0.81	80.5	74.3	75.1	80.1
KNN	62.7	0.67	79.6	45.7	48.1	91.1
SVM	77.0	0.79	80.8	73.2	74.6	79.0
Naive Bayes	**78.8**	**0.82**	**82.0**	**75.5**	**75.9**	**81.6**

AUC, area under the curve; PPV, positive predictive value; NPV, negative predictive value; KNN, k-nearest neighbors; SVM, support vector machines. Bold values indicates best-performing algorithm.

**Table 3:** Final Naïve Bayes model performance measures used on the validation dataset.

Performance measures	% (95% confidence intervals)
Balanced accuracy	78.2 (72.1–82.9)
AUC	0.82 (0.75–0.87)
Sensitivity	80.7 (72.2–87.1)
Specificity	75.7 (66.1–83.4)
PPV	79.3 (70.8–85.9)
NPV	77.2 (67.6–84.7)

**Table 4:** Absolut and relative distribution of relevant predictor variables.

Variable description	Forensic psychiatric patients *n*/*N* (%)	General psychiatric patients *n*/*N* (%)
Type of delusion: bizarre	**155/337 (46.0)**	7/354 (2.0)
Delusion: aggressive content	**234/338 (69.2)**	66/351 (18.8)

Bold values indicates most frequent occurrence.

**Figure 2:**
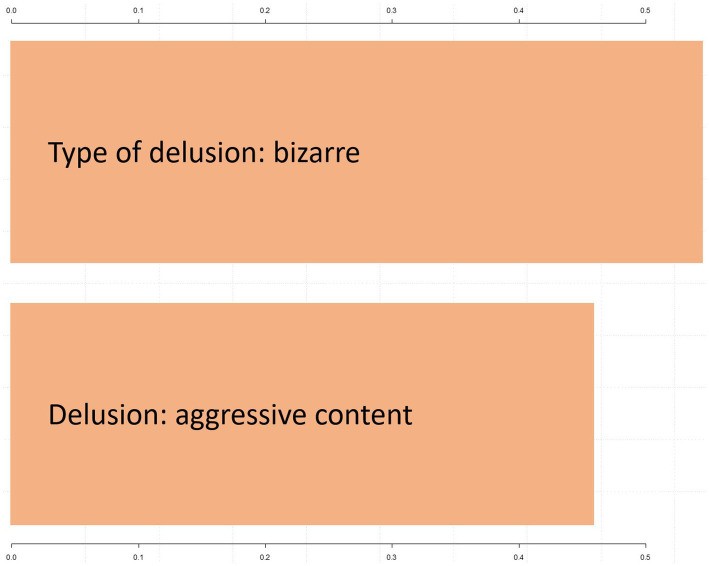
Predictor variables’ relative influence to the model. Both bizarre delusions and aggressive delusional content contribute substantially to discriminating between forensic and general psychiatric patients, highlighting the importance of systematically assessing both characteristics during clinical risk evaluations.

The model training revealed two influencing variables. First, bizarre delusions occurred in nearly half of FPP, while these were rather neglectable in GPP (46% vs. 2%). They were defined as delusions that are clearly implausible and not understandable to same-culture peers and do not derive from ordinary life experience (APA, 2013). Second, FPP more often suffered from aggressive delusional content (69% vs. 19%). Aggressive delusional content was defined as delusions containing themes of aggressive nature documented in patient files. This included delusions where aggression was thematically central to the delusional belief, regardless of whether the perceived aggression was directed toward the patient, originated from the patient, or involved actual criminal behavior (as in FPP). Aggression was defined as “behavior intended to harm another person who is motivated to avoid that harm” (Allen and Anderson, 2017). Notably, none of the remaining 101 items, e.g., regarding PANSS, appeared to be influential. Table 4 and Figure 2 display the two variables’ absolute as well as relative distribution and relative influence, respectively.

To further assess the robustness of the identified predictors, the distribution of bizarre delusions and aggressive delusional content was examined separately in the training and validation datasets (see Supplementary Table S2). The observed group differences were highly consistent across both subsets, with both variables remaining substantially more prevalent in forensic psychiatric patients compared to general psychiatric patients. Only minor variations in absolute frequencies were observed, supporting the stability and generalizability of these predictors beyond the training data.

## 4 Discussion

In the present study, bizarre delusions (46% of FPP vs. 2% of GPP) and aggressive delusional themes (69% of FPP vs. 19% of GPP) were key differentiating factors in the ML model when distinguishing between FPP und GPP. The model showed a good level of discrimination as indicated by an AUC of 0.81 and a balanced accuracy of 78.2% (Nahm, 2022). Since these delusional themes were more prevalent in the forensic setting, the findings provide empirical support for the discriminative value of bizarre and aggressive delusions and reinforce the notion that certain delusional themes warrant heightened clinical attention. The results align with prior studies emphasizing the role of persecutory delusions, where the individual believes they are being harmed or persecuted by others, and command hallucinations, which have shown strong associations with aggressive actions (Bjørkly, 2002) (Cheung et al., 1997; Coid et al., 2013; Keers et al., 2014; Lamsma and Harte, 2015). The present findings are consistent with the hypothesis that delusional content is phenomenologically relevant in the context of offending behavior in SSD. It must be acknowledged, however, that the retrospective design precludes any determination of whether these delusional themes preceded the offense or emerged thereafter – whether as post-hoc rationalization, because of the forensic context itself, or through reinforcement during prolonged inpatient treatment. Also, our findings may be interpreted in the context of the threat/control-override (TCO) theory, which posits that perceived threats and loss of self-control precipitate violent actions in SSD patients (Stompe et al., 2004). Previous research revealed that persecutory ideations are associated with aggressive behavior, particularly when accompanied by elevated levels of delusional distress, suggesting that emotional responses to perceived threat may play an important role in symptom-consistent aggression (van Dongen et al., 2012b; van Dongen et al., 2012a). In the present study, delusional categories were not coded as mutually exclusive. Consequently, persecutory beliefs involving themes of violence, poisoning, torture, assault, or other forms of harm could simultaneously be coded as persecutory delusions and as aggressive delusional content. Our findings therefore do not contradict the existing literature on persecutory ideation and threat-related mechanisms. Rather, they suggest that aggression-related themes within delusional beliefs may represent a broader phenomenological characteristic that is overrepresented in forensic psychiatric patients with SSD.

However, the study also challenges some of the existing literature that report inconsistent links between specific delusional themes and violence. For instance, Douglas et al. found no significant connection between delusional themes and aggression, suggesting that other factors might play a more substantial role. The discrepancies in findings may be attributed to methodological differences, such as variations in sample size, definitions of violence, and the context in which delusions occur (Douglas et al., 2009). Other important literature that found no association between violence and delusions include the MacArthur Violence Risk Assessment Study, which covered a one-year follow-up period after hospitalization. The authors stated that controlling for impulsiveness and anger annihilated the particular risk of violent actions (Appelbaum et al., 2000). Other studies conclude that violence is attributed to delusional content in only a minor part of patients (Junginger et al., 1998).

These findings collectively underscore that delusional content still must be interpreted in a broader context. Established risk factors for violence in SSD – including substance use disorders, medication non-adherence, impulsivity, anger dysregulation, and social marginalization – account for a substantial proportion of violence risk and must remain central to any comprehensive forensic risk assessment (Swartz et al., 1998; Fazel et al., 2009b; Bo et al., 2011; Witt et al., 2013). The present findings suggest that specific delusional themes may constitute a clinically relevant additional dimension, but one that is best understood as part of a multifactorial framework rather than as a standalone marker. This is particularly relevant since the majority of individuals with SSD never engage in violent behavior, and that population-level associations between psychopathological features and offending do not translate directly into individual-level risk (Whiting et al., 2022).

Some bizarre delusions – which were in general found to be model defining in the present study – have been understood as possible drivers of violence before. In particular, the delusional perception of relatives being replaced by impostors are linked to severe aggression (Nestor et al., 1995; Bourget and Whitehurst, 2004; Green et al., 2009; Young, 2024). Moreover, previous research showed that psychotic symptoms that are associated with anger may lead to violent behavior, especially in persecutory delusions (Ullrich et al., 2013; Keers et al., 2014). While our study did not directly include affects related to delusions (rather affect accompanying illness), anger might still be part of psychopathology in FPP considering the high presence of delusions with aggressive content in this group. This is particularly important since existing evidence focuses on anger as a primary treatment target in order to reduce the risk of violence (Ullrich et al., 2018).

Additionally, our findings contribute to the ongoing debate regarding the relationship between grandiose delusions and violence: While studies such as Witt et al. suggest that grandiose delusions can lead to aggressive acts due to perceived entitlement, our study did not identify grandiose delusions as a distinguishing factor between FPP and GPP (Witt et al., 2013). This discrepancy highlights the complexity of violent behavior in SSD patients and suggests that further research is needed to clarify these associations.

Importantly, our study also corroborates the emotional response theory, where delusions associated with anger, frustration, and fear, such as the belief that one is being persecuted, often mediate violent behavior. A key finding is that anger caused by delusional thinking is a crucial factor explaining the link between violence and psychosis. Despite the low percentage of violent acts caused by mental illness, evidence consistently supports a connection between delusions and violence. This is consistent with studies highlighting that emotional distress generated by delusions often catalyzes violent behavior (Giorgos, 2017; Grohmann et al., 2024).

It has to be mentioned that none of the PANSS items (neither sum score nor specific symptoms upon admission and discharge) contributed to the ML model, indicating comparable baseline symptom severity between both groups. On the one hand, this poses a methodological strength as both FPP and GPP exhibit comparable overall psychopathology. On the other hand, this similarity provides a clinically important insight: the specific content of delusions (bizarre and aggressive themes) appears to be critical for forensic outcomes. Since the PANSS serves as a vital clinical tool to assess symptom severity, we suggest complementing psychometric evaluations with specific evaluation of delusional themes during clinical interviews rather than replacing these. However, the markedly longer treatment duration in forensic psychiatric settings represents an important contextual factor when interpreting the findings. Extended observation periods in FPP may increase the likelihood of detecting and documenting specific delusional themes, whereas shorter inpatient stays in GPP may restrict the observation of less overt or fluctuating symptoms. While the observed differences in symptomatology content were substantial, this potential detection bias should be considered when interpreting group differences.

The present findings also complement an earlier study (Grohmann et al., 2024), which examined delusional content within a forensic psychiatric sample exclusively, comparing violent and non-violent offenders with SSD. While Grohmann et al. found that aggressive delusional content and associated negative affect were associated with violent offending within the forensic group, the present study demonstrates that the same variable discriminates between FPP and GPP, i.e., between those who have and have not entered the criminal justice system. These convergent findings from two complementary analytical perspectives suggest that aggressive delusional content may represent a clinically meaningful phenomenological marker at multiple levels of forensic relevance, while the distinct research questions, samples, and outcome variables preclude direct comparison of results.

From a clinical perspective, these findings underline the importance of systematically assessing not only the presence of psychotic symptoms but also their qualitative content. Clinicians should place significant emphasis on the content of delusions during their evaluations, especially looking for bizarre and aggressive delusional themes, as these were found to differ substantially between FPP and GPP diagnosed with SSD. Recognition of these delusional themes could aid in the formulation of personalized treatment plans and systematic assessment of delusional content may complement standard psychometric tools such as the PANSS. These findings further suggest that ML based approaches may offer a useful framework for identifying phenomenologically relevant variables in forensic psychiatric research, though the feasibility of their integration into clinical risk assessment practice has yet to be shown using prospective validation in independent samples.

### 4.1 Limitations

While the study offers valuable insights into the relationship between delusional content and offending behavior in SSD, several limitations must be acknowledged. The retrospective design of the study limits the ability to establish causality. Importantly, we cannot determine whether the identified delusions preceded the criminal behavior or developed afterward, potentially as a post-hoc rationalization or during the course of treatment. This temporal ambiguity limits our ability to make predictions about future offending risk based on current delusional content. In addition, the outcome variable of the present study was group affiliation (forensic versus general psychiatric treatment setting), rather than the occurrence of criminal or violent behavior itself. Consequently, the findings should be interpreted as identifying differences in the thematic expression of delusions between these clinical populations, rather than as evidence that specific delusional contents directly predict offending. Any interpretation in terms of risk or criminal behavior must therefore remain cautious and contextualized.

Furthermore, the definition of forensic patients inherently includes having committed an offense, which may create a relationship with violence-related delusions. This circularity constrains the applicability of our findings for predicting future offending in non-forensic populations. Additionally, not all individuals with SSD who commit offenses enter forensic psychiatry; some may be imprisoned without psychiatric treatment or receive only outpatient care. Our sample may therefore represent only the most severely ill offenders, introducing selection bias (Fazel et al., 2009a).

A further limitation concerns the structural asymmetry in documentation sources between groups: whereas FPP records routinely include police and legal reports, GPP documentation was limited to clinical records. This creates a risk of ascertainment bias, whereby delusional themes may be more thoroughly recorded in FPP due to the demands of the legal evaluation context rather than genuine psychopathological differences. Notably, however, such a bias would be expected to elevate the recorded prevalence of delusional themes broadly across FPP, rather than selectively – a pattern not observed in our data, where the majority of assessed themes showed no meaningful group difference. The reliance on electronic health records means that the quality of the data is dependent on the accuracy and completeness of the documentation, which likely varied across the 34-year study period. While interrater reliability is considered substantial (Brennan and Hays, 1992) (Cohen’s Kappa 0.78, following the independent review of a random 10% dataset subsample by a second rater), the coding of delusional content, particularly for the subjective assessment of “bizarreness,” may be culturally influenced and vary between raters (Cermolacce et al., 2010). Additionally, the study period may have introduced inconsistencies in documentation practices. However, the sensitivity analysis restricting data to 2000–2016 revealed an AUC and balanced accuracy within the confidence intervals of the full analysis. This suggests that temporal variations did not substantially compromise our findings. Moreover, while the initial variable space of 103 predictors was large relative to the sample size, dimension reduction to two final predictors substantially mitigates overfitting concerns. The combination of nested cross-validation, a held-out validation set, multiple applied ML techniques and sensitivity analyses, and temporal stability supports the robustness of the core findings, though external replication is considered necessary before broader generalizations can be drawn.

Furthermore, the sample is drawn from a single Swiss institution, which limits the generalizability of the findings to other – especially legal – settings or populations. As the study focuses on psychopathological themes, it does not capture the full complexity of factors contributing to violent behavior in SSD patients, particularly among women, who are underrepresented in the sample. We also did not differentiate between types of violence (e.g., reactive vs. instrumental), severity of offenses, or offender subtypes such as early- and late-starting offending trajectories (Hodgins et al., 2014; Van Dongen et al., 2015), as the available sample size would not have allowed a reliable analysis without a substantial risk of unstable estimates and overfitting. Accordingly, we cannot determine whether aggressive delusional content and bizarre delusions are equally relevant across all offender subgroups or whether their discriminative value is stronger in specific trajectories or types of offenders with SSD. Future studies using larger forensic samples and developmental offending data should examine whether delusional content contributes differently across these subgroups.

Last, as seen in Table 1, FPP exhibited a higher prevalence of substance use disorders compared to GPP (73% vs. 56%). As substance use is a widely known mediating factor of violent behavior in SSD, we had not been able to rule out an confounding influence (Fazel et al., 2009b). Since the present study strictly focused on symptomatology, possible risk factors like substance use and personality disorders were not included as primary predictors. A *post hoc* sensitivity analysis including both comorbid substance use and personality disorder as additional predictors yielded only minimal model changes. No causal inferences regarding violent behavior can however be drawn from this retrospective design.

### 4.2 Conclusion

This study highlights the significant role of delusional content, particularly bizarre and aggressive delusions, in differentiating between forensic and general psychiatric patients diagnosed with SSD. These findings underscore the importance of detailed assessment and management of delusional content in clinical practice. While aligning with some existing literature, the findings also challenge prior assumptions and underscore the need for further research to unravel the complexities of delusions and offending in SSD. By addressing these gaps, future research may contribute to more effective interventions and support systems for this vulnerable population.

## Data Availability

The original contributions presented in the study are included in the article/Supplementary material, further inquiries can be directed to the corresponding author.
