# Supplementary material for: Delusional themes and forensic status in schizophrenia spectrum disorders: a machine learning based discriminative study
Source: Front Psychol. 2026 Jul 22;17:1840906. doi: 10.3389/fpsyg.2026.1840906 (PMC13437467; doi:10.3389/fpsyg.2026.1840906)
Supplement: Supplementary file 1 [file Table_2.DOCX]

Supplementary Table S2

| **Dataset** | **Training Dataset** | | **Validation Dataset** | |
| --- | --- | --- | --- | --- |
| **Variable description** | **FPP**  **n/N (%)** | **GPP**  **n/N (%)** | **FPP**  **n/N (%)** | **GPP**  **n/N (%)** |
| Type of delusion: bizarre | **116/246 (47.2)** | 3/242 (1.2) | **39/91 (42.9)** | 4/112 (3.6) |
| Delusion: aggressive content | **164/245 (66.9)** | 46/240 (19.2) | **70/93 (75.3)** | 20/111 (18.0) |

**Supplementary Table S2.** Distribution of bizarre delusions and delusions with aggressive content in the training and validation dataset.
FPP = Forensic psychiatric patients, GPP = General psychiatric patients.
